# Prehospital Detection of a Large Pericardial Effusion Using Screening Sonography: A Case Report

**DOI:** 10.7759/cureus.98471

**Published:** 2025-12-04

**Authors:** Sergio Miravent, Juan M Ruiz, Narciso B Molina, Carmen J Bermejo, Catarina M Mascarenhas

**Affiliations:** 1 Basic Emergency Service, Algarve Local Health Unit, Vila Real de Santo António, PRT; 2 Medical Imaging and Radiotherapy, School of Health, University of the Algarve, Faro, PRT

**Keywords:** emergency, pericardial effusion, screening, tamponade, ultrasound

## Abstract

Pericardial effusion, although not uncommon in emergency contexts, poses detection and diagnostic challenges in prehospital and peripheral emergency settings where access to advanced imaging and specialist evaluation is limited. In its severe forms, rapid fluid accumulation may lead to cardiac tamponade, a life-threatening condition requiring immediate recognition and intervention. The clinical course is often insidious, and the progression of symptoms may easily be mistaken for other, more common pathologies, further complicating timely diagnosis. We report the case of a patient who presented twice within two days to a Basic Emergency Service with central chest pain of five days’ duration, low-grade fever, and subsequently worsening dyspnea. Given the persistence and evolution of symptoms, a bedside screening ultrasound was performed, which revealed a large pericardial effusion. The patient was urgently referred to a central hospital, where the diagnosis was confirmed, and appropriate management was instituted. This case illustrates the vital function of screening ultrasound in resource-limited environments: even when definitive treatment cannot be provided locally, early identification of critical findings supports timely referral and improves patient outcomes. Furthermore, the decision to employ or omit screening echocardiography in such contexts can significantly influence clinical pathways and ultimately alter patient prognosis, emphasizing its relevance as a low-cost, rapidly deployable screening tool, while also stressing that patient outcomes may depend on the availability of a trained sonographer/radiographer capable of performing the examination.

## Introduction

Pericardial effusion covers a wide spectrum of presentations, ranging from incidental findings on imaging to rapidly progressive, life-threatening tamponade. In emergency care, its recognition is particularly demanding because the condition often lacks specific signs [1], and conventional diagnostic tools such as electrocardiography or chest radiography provide limited sensitivity. The electrocardiogram (EKG) may show electrical alternans or low QRS voltage in multiple leads in the setting of large effusions or tamponade, but these findings are suboptimal for diagnostic purposes [2]. For chest radiography to depict a pericardial effusion, more than approximately 200 mL of fluid is typically required, so smaller yet clinically relevant effusions can remain occult [3].

Diagnostic uncertainty contributes to a delay in the detection of pericardial effusions; many remain undiagnosed during initial evaluation and are only discovered once patients deteriorate or advanced imaging is performed [4]. In peripheral and prehospital services, where access to cardiology consultation and echocardiography is not immediate and requires transfer to a referral hospital nearly an hour away, the challenge is even greater.

Screening sonography offers a pragmatic solution: it can rapidly confirm the presence of fluid in the pericardial sac. Clinically, pericardial effusion often manifests with nonspecific complaints such as precordial chest pain, exertional dyspnea, low-grade fever, or generalized malaise, making early recognition challenging. As effusion volume increases or when fluid accumulates rapidly, the clinical picture may shift toward signs of hemodynamic compromise, including tachycardia, hypotension, jugular venous distension, and pulsus paradoxus. These findings define the transition to cardiac tamponade, which remains a clinical diagnosis [5]. Identification of fluid within the pericardial cavity, which typically appears as an anechoic (black) image surrounding the heart and creates a measurable echo-free space (EFS) between the visceral leaflet and parietal leaflet [6,7].

The size of the effusion can range from small (<10 mm EFS), moderate (10-20 mm EFS), large (>20 mm EFS), and massive (>25 mm EFS). However, the severity of a pericardial effusion is not determined solely by its volume but also by the rate at which the fluid accumulates. Rapid accumulation may not allow the pericardium enough time to stretch and accommodate the fluid, leading to an increased risk of cardiac tamponade [8].

Although cardiac ultrasound screening is not intended to replace formal diagnostic examinations, it remains the case that through eyeballing - a subjective visual assessment of cardiac function and structures from multiple imaging planes [9] - ultrasound provides a more detailed evaluation of whether the pericardial effusion is small and potentially chronic, possibly associated with underlying pathologies, or whether the fluid surrounds the entire heart and exerts pressure on the cardiac chambers to the point of causing tamponade. Although tamponade is a clinical diagnosis, certain hallmark echocardiographic signs can strongly suggest its presence. These include pericardial effusion, diastolic collapse of the right ventricle (more than 90% specific for tamponade), right atrial collapse during systole (lasting more than one-third of the cardiac cycle), a plethoric inferior vena cava, and significant respiratory variation in mitral and tricuspid inflow velocities on pulse wave Doppler from the apical view (sonographic pulsus paradoxus). It's important to note that tamponade can occur with varying degrees of pericardial effusion [10].

Published evidence indicates that screening ultrasound emerged to address a practical unmet need, an operational gap at the moment of clinical decision-making, when information is incomplete and time is critical. Pericardial effusion is a typical example of where screening ultrasound performed by non-specialist physicians or other healthcare professionals can have immediate clinical value, because it reduces the diagnostic gap between suspicion and confirmation through a differentiated, real-time imaging modality that enables direct bedside assessment of the effusion and its physiological impact. This increases diagnostic certainty with few false negatives, supports safer decision-making, and accelerates time-critical management, particularly in acute and resource-limited emergency settings.

## Case presentation

**Figure 1:**
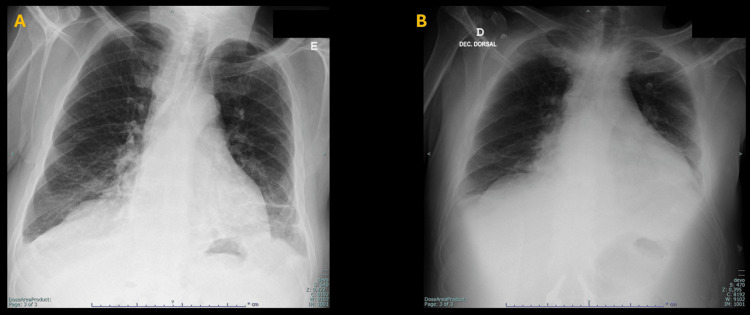
There is a two-day interval between chest X-rays A and B from the same patient. Image panel A represents an orthostatic posteroanterior chest X-ray that reveals a subtle increase in the cardiac silhouette and mild costophrenic sinus effacement bilaterally. Image panel B is a decubitus anteroposterior chest X-ray obtained two days after the chest image in panel A, and demonstrates significant progression, with a marked increase in the cardiac silhouette and more pronounced bilateral costophrenic sinus effacement.

A 54-year-old male patient presented to a Basic Emergency Service (BES), brought by his sister due to central chest pain for approximately five days and a history of dyspnea for the past three days. Two days earlier, he was attended to the same BES and was classified as Urgent (yellow) according to the Manchester Triage System (MTS). On that occasion, an orthostatic chest X-ray (Figure 1A) and an EKG were performed, with no significant abnormalities reported. A snippet of the physician's report was, "…patient has bilateral lower limb edema with no signs of deep vein thrombosis (DVT). Treatment is symptomatic. A follow-up with a family doctor is recommended in 48-72 hours if there is no improvement or sooner to reference hospital if the condition worsens. Heart failure is under investigation….”

The patient returned to BES with additional complaints of vomiting and numbness in the lower limbs, which began that afternoon. His priority classification was again marked as Urgent (yellow) according to MTS. During this episode, his vital signs included a tympanic temperature of 36.1°C. His Glasgow Coma Scale (GCS) score was 15. His oxygen saturation in room air was 91%, with a heart rate of 112 beats per minute, with a blood pressure of 105/65 mmHg.The doctor requested a thoracic X-ray (Figure 1B).

The radiographer, upon reviewing the chest X-ray obtained immediately beforehand, proceeded to perform a screening ultrasound using the RUSH protocol (Rapid Ultrasound in Shock) [11] to enable a rapid global assessment of shock or other acute causes not yet clinically clarified. The examination addressed the core RUSH components: the “pump” (focused cardiac assessment), the “tank” (inferior vena cava evaluation and a Focused Assessment with Sonography for Trauma (FAST)-style abdominal survey for free fluid), and the “pipes” (focused assessment of the abdominal aorta). Dedicated lung views were not performed, as a chest X-ray had just been acquired moments earlier. The cardiac assessment was obtained primarily through the subxiphoid (SUBX) window, which represents an acceptable practical adaptation of the protocol when other windows are limited. The survey was performed predominantly with a standard abdominal curvilinear probe, while the cardiac windows were acquired using a phased-array (sector) cardiac transducer; findings are succinctly summarized through three videos and two still images.

During cardiac windows acquisition, a pericardial effusion is detected and further assessed using the SUBX and apical four-chamber views (A4CV), which are displayed in Videos 1A-1B.

**Video 1:** Cardiac ultrasound panels A and B via subxiphoid incidence and apical four-chamber view (RUSH protocol). This video illustrates cardiac imaging in subxiphoid view (SXV) perspective in panel A, and panel B depicts a four-chamber view (A4C). Key structures are identified, including the left ventricle (LV), left atrium (LA), right ventricle (RV), and right atrium (RA). Additionally, pericardial effusion (PE) is visible as the black area surrounding the heart, marked with an asterisk (*). RUSH: Rapid Ultrasound in Shock

Complementary parasternal long axis (PLAX) and parasternal short axis views (PSAX) are also acquired and displayed in Videos 2A-2B. The bilateral pleurodiaphragmatic transitions study also confirmed bilateral pleural effusions, which were displayed in Videos 3A-3B.

**Video 2:** Biplanar parasternal long- and short-axis views of the heart in panels A and B, respectively. Panels A and B display a parasternal long-axis (PLAX) view and a short-axis view (PSAX) at the level of the papillary muscles, respectively. Here, key structures are identified, including the left ventricle (LV), left atrium (LA), right ventricle (RV), and right atrium (RA). Additionally, pericardial effusion (PE) is visible as the black area surrounding the heart, marked with an asterisk (*). AR: aortic root

**Video 3:** Right and left pleurodiaphragmatic transitions in panels A and B, respectively. Pleurodiaphragmatic transitions are visualized, with a purely anechoic zone above the diaphragm corresponding to bilateral pleural effusions. On the right side (panel A), there is associated gallbladder wall thickening, a common finding in heart failure. PE: pleural effusion, LI: liver, LU: lung, SP: spleen, GB: gallbladder, LK: left kidney

**Figure 2:**
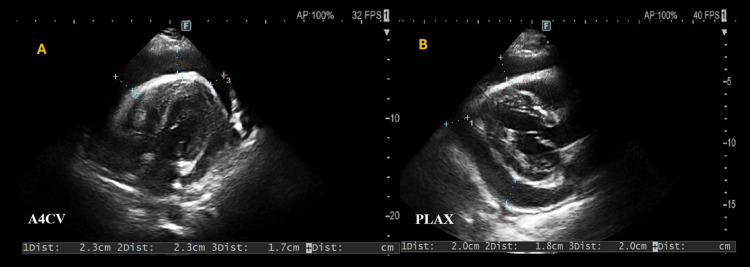
EFS measurements in the A4C and PLAX cardiac incidences in panels A and B, respectively. Panels A and B show still images acquired in the apical four-chamber (A4C) and parasternal long-axis (PLAX) views, respectively, with measurements of echo-free space (EFS) of approximately 2 cm, consistent with a large pericardial effusion.

Measurements of pericardial effusion are displayed in Figures 2A-2B, which represent A4CV and PSAX planes. In the remaining examination, assessment of the inferior vena cava demonstrated a diameter of 2.1 cm with respiratory variation, and the abdominal aorta was considered normal; bilateral superior and lower recesses presented without free fluid.

All blood tests available at BES were required and obtained. Arterial blood gas analysis showed pH: 7.38 (normal: 7.35-7.45), pCO_2_: 23 mmHg (normal: 32-48 mmHg), pO_2_: 76 mmHg (normal: 83-108 mmHg), lactate: 7.6 mmol/L (normal: 0.5-2.0 mmol/L), HCO_3_^-^: 16 mEq/L (normal: 22-26 mEq/L), and base excess (BE): -11 (normal: -2 to +2). Blood tests indicated hemoglobin (Hb): 10.7 g/dL (normal: 13.5-17.5 g/dL), hematocrit (Hct): 31% (normal: 38-50%), and platelets (Plaq): 1375000/µL (normal: 150,000-450,000/µL), with a normal white cell series. EKG revealed possible atrial fibrillation with a ventricular rate of 110 bpm and low voltage; cardiac troponin levels were negative. The doctor on duty, after analyzing all available data and relying heavily on real-time ultrasound images, implemented carefully supportive fluid therapy and expedited the patient's transfer to the reference hospital. Approximately an hour later, the patient arrived at the referral hospital in agonal respiration and, in the emergency room, went into cardiac arrest. Cardiopulmonary resuscitation was initiated, and emergency pericardiocentesis was performed, draining over 600 mL of pericardial fluid. The patient was successfully resuscitated and remained in intensive care.

**Figure 3:**
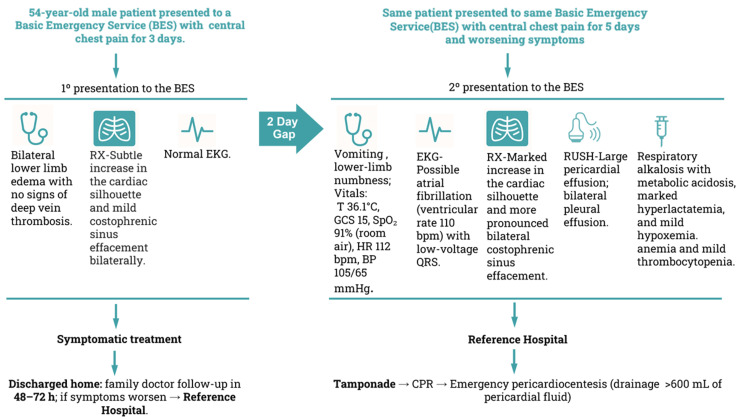
Resumed timeline of local care settings and clinical findings of the case report. Timeline outlining the local care settings (BES and reference hospital) and the main clinical findings over two presentations, integrating EKG, chest X-ray, RUSH findings, and laboratory results, with progression to cardiac tamponade requiring CPR and emergency pericardiocentesis (drainage >600 mL). bpm: beats per minute, BP: blood pressure, CPR: cardiopulmonary resuscitation, EKG: electrocardiogram, GCS: Glasgow Coma Scale, HR: heart rate, RUSH: Rapid Ultrasound in Shock, SpO_2_: peripheral capillary oxygen saturation

Figure 3 presents a concise timeline of the events in this case report, summarizing the clinical course and the care settings/locations, along with the key clinical, laboratory, and imaging findings at each time point.

## Discussion

This case illustrates the value of screening ultrasound in identifying a large pericardial effusion in a prehospital environment, where clinical tools are scarce, and typical signs may be subtle or misleading.

In first attendance at the BES, the patient presented with central chest pain, shortness of breath, and bilateral lower limb edema, features that can also be associated with pericardial effusion [12]. Initial assessments, including chest X-ray and EKG, showed no alarming findings. This reflects a familiar issue: early pericardial effusion, or even evolving tamponade, often escapes detection when symptoms develop gradually and lack specificity [13]. However, when reviewing the first chest radiograph obtained at the patient’s initial access to the BES, one cannot exclude that a pericardial effusion was already present, since a careful observation revealed a subtle increase in the cardiac silhouette and small bilateral pleural effusions. In the context of precordial pain, this should have raised the possibility of an associated pericardial effusion from the outset.

What altered the course of this case was a focused ultrasound, performed promptly by a radiographer trained in emergency sonography. Even for the non-cardiology clinician, a quick visual assessment of cardiac motion and chamber behaviour can provide crucial information. Indicators such as right chamber compression, distended inferior vena cava, and respiratory variation in ventricular filling may suggest that hemodynamic compromise may occur.

Ultrasound made a large pericardial effusion apparent in this case, but unequivocal signs of cardiac tamponade were not demonstrated. While direct evidence of right-sided chamber collapse was unclear, a subtle impairment of ventricular diastolic filling cannot be excluded. Taken together with the clinical picture, these findings raise concern for impending tamponade.

Comparison of the two chest radiographs obtained, however, suggested that the accumulation of pericardial fluid occurred rapidly, a circumstance often associated with the potential for tamponade. This interpretation proved consistent with the patient’s clinical course, as cardiac tamponade with consequent obstructive shock developed approximately one hour after leaving the BES and upon arrival at the referral hospital. Laboratory results from the BES also revealed signs of tissue hypoperfusion, including elevated lactate, low bicarbonate levels, a relatively low blood pressure profile, and an EKG with low-voltage QRS complexes. These findings, together with the ultrasound images, reinforced the clinical suspicion and justified the urgent transfer to the referral hospital. The platelet value was most likely a transcription or analytical machine error, with the clinically plausible result being mild thrombocytopenia rather than true thrombocytosis, considering that the only known comorbidity was treated hypothyroidism.

Chest radiographs have long been shown to lack sensitivity in detecting pericardial effusion [14], which leads to a relevant question: why isn’t screening ultrasound used more routinely in frontline emergency settings? Despite being quick, non-invasive, and highly informative, it remains underused [15].

Although pericardial effusion often presents with subtle or nonspecific findings, recognition is strongly dependent on timely echocardiography. In a retrospective academic-center cohort study of patients requiring pericardiocentesis, point-of-care ultrasonography proved to shorten time to diagnosis (mean 5.9 hours vs. >12 hours with other imaging) and time to pericardiocentesis (28.1 hours vs. >48 hours), while physical examination, chest radiography, and EKG were frequently non-diagnostic [16]. European consensus documents from the European Association of Cardiovascular Imaging (EACVI) reaffirm echocardiography as the preferred and most accurate modality to detect effusion and assess hemodynamic impact in the acute setting, underscoring the risk of underdiagnosis where echocardiography is not promptly available [17].

Pericardial effusion is not rare, and without early recognition, it can quickly progress to a life-threatening scenario. Here, pericardiocentesis is the only procedure that can effectively save the patient. Therefore, the liberal use of bedside ultrasound is strongly recommended, particularly for patients presenting with shortness of breath and chest pain [18,19]. Even without detailed knowledge of cardiac anatomy or function, a simple SUBX view is often enough to detect a pericardial effusion, showing how basic ultrasound skills can make a critical difference in emergency settings. In situations where time is limited and human and technological resources are stretched, tools with direct impact on patient outcomes should take priority [20].

Screening ultrasound is not a substitute for specialist evaluation and has important technical limitations, chief among them its reliance on operator skill. Nevertheless, when higher education systems invest in university-trained radiographers with competencies in screening ultrasound, not integrating this workforce into healthcare delivery, even in well-established, protocol-driven, lifesaving applications, creates a paradox in resource allocation. Such underutilization delays the diagnostic process and ultimately represents a missed opportunity to reduce both the human burden of late detection and the technical and material costs associated with it.

## Conclusions

Screening ultrasound supports frontline clinicians in resource-limited emergency settings to act with greater clarity and speed. Its use at the bedside can transform initial uncertainty into targeted, lifesaving interventions, as demonstrated by the early detection of pericardial effusion that standard tools might have overlooked. By integrating clinical judgment with real-time imaging, patient stabilization and timely referral become achievable even in peripheral services. The barriers to widespread adoption, whether insufficient training, limited access to portable equipment, entrenched clinical habits, or restrictive legal frameworks, must be acknowledged and actively addressed. Persisting in models that underutilize trained professionals capable of performing protocolized lifesaving ultrasound is both a paradox in resource allocation and a missed opportunity to improve outcomes.

Clinicians in basic emergency settings should therefore adopt echocardiography in the routine evaluation of patients presenting with chest pain and dyspnea, as consistently recommended in the literature. Bedside ultrasound is not an optional add-on; it is an essential tool for modern emergency care, and its systematic integration should be regarded as a priority for health systems committed to equity, safety, and timely diagnosis.
